# Retraction: Management practice, and adherence and its contributing factors among patients with chronic kidney disease at Tikur Anbessa Specialized Hospital: A hospital-based cross-sectional study

**DOI:** 10.1371/journal.pone.0328178

**Published:** 2025-07-14

**Authors:** The PLOS One Editors

This study [1] used an adapted version of the Morisky Medication Adherence Scale (MMAS-8) scale [2,3].

The article [2] reporting the psychometric properties and validation of the MMAS-8 scale was retracted in 2023 [3] due to concerns regarding the sensitivity and specificity of the medical adherence scale and the reliability of the article’s conclusions. The retraction of the scale’s validation study calls into question the validity and reliability of results obtained using the MMAS-8, including adapted and translated versions.

Given the reliance of this article’s [1] study design on the MMAS-8, the *PLOS One* Editors retract this article due to concerns about the reliability of the reported results and conclusions.

All authors either did not respond directly or could not be reached.

Note: After this article [1] was published, concerns were raised that the same work was published shortly after in the *International Journal of Nephrology* [4]. The article was submitted to both journals on the same date, which does not comply with PLOS policy. According to the corresponding author, this resulted from a communication issue within the author group. The second article [4] was retracted in February 2019 [5] due to this dual publication issue.
